# The importance of comprehensive diagnostic work-up and genetic testing to reveal Andersen–Tawil syndrome—a case report

**DOI:** 10.1093/ehjcr/ytag064

**Published:** 2026-01-31

**Authors:** Dorte Stavnem, Priya Bhardwaj, Jacob Tfelt-Hansen, Carl Johann Hansen, Bo Gregers Winkel

**Affiliations:** Department of Cardiology, The Heart Centre, Copenhagen University Hospital, Rigshospitalet, Blegdamsvej 9, 2100 Copenhagen, Denmark; Department of Cardiology, The Heart Centre, Copenhagen University Hospital, Rigshospitalet, Blegdamsvej 9, 2100 Copenhagen, Denmark; Section of Forensic Genetics, Department of Forensic Medicine, Faculty of Health and Medical Sciences, University of Copenhagen, Frederik V's Vej 11, 2100 Copenhagen, Denmark; Department of Cardiology, The Heart Centre, Copenhagen University Hospital, Rigshospitalet, Blegdamsvej 9, 2100 Copenhagen, Denmark; Section of Forensic Genetics, Department of Forensic Medicine, Faculty of Health and Medical Sciences, University of Copenhagen, Frederik V's Vej 11, 2100 Copenhagen, Denmark; Department of Cardiology, The Heart Centre, Copenhagen University Hospital, Rigshospitalet, Blegdamsvej 9, 2100 Copenhagen, Denmark; Department of Cardiology, The Heart Centre, Copenhagen University Hospital, Rigshospitalet, Blegdamsvej 9, 2100 Copenhagen, Denmark

**Keywords:** Andersen-Tawil syndrome, Ventricular ectopy, Ventricular arrhythmias, Sudden cardiac arrest, Implantable cardioverter-defibrillator, Case report

## Abstract

**Background:**

Andersen–Tawil syndrome is characterized by a symptom triad of cardiac electrical abnormalities, periodic muscular paralysis, and distinct dysmorphic manifestations. A history of unexplained syncope has been associated with a more serious phenotype with increased risk of life-threatening arrhythmia. Due to the syndrome’s rarity and highly variable clinical presentation, diagnosis remains challenging.

**Case summary:**

This report highlights the importance of comprehensive diagnostic workup following a sudden cardiac arrest, particularly emphasizing the value of genetic testing. We present a 61-year-old male hypertensive patient who initially presented with a first-time syncopal episode. Initial investigations revealed ventricular ectopy exceeding 12 000 premature ventricular contractions, occasional QT prolongation of >500 ms, and mildly reduced left ventricular ejection fraction (50%). Outpatient diagnostic investigations did not yield a diagnosis. While awaiting ablation, the patient suffered from an out-of-hospital cardiac arrest and was successfully resuscitated after 17 min. Complete diagnostic work-up including guideline-adherent assessments and genetic testing eventually revealed Andersen–Tawil syndrome. The subsequent family evaluations supported the diagnosis.

**Discussion:**

Diagnosis was unexpected as the patient presented with isolated cardiac manifestations and a late onset of symptoms. Cardiomyopathy and primary arrhythmic disorders were relevant differential diagnoses and investigated during admission. No clinical assessment is pathognomonic for Andersen–Tawil syndrome, making genetic testing essential for establishing a definitive diagnosis. While historically characterized as a long QT variant, research suggests Andersen–Tawil syndrome is its own disease entity. Pharmacological management follows established channelopathy principles, though the protective efficacy of beta-blockers and flecainide remains uncertain in this syndrome.

Learning points

- Comprehensive diagnostic work-up including genetic testing is crucial for diagnosing rare causes of cardiac arrest like Andersen–Tawil syndrome.
- Unexplained multifocal ventricular ectopy and prominent U waves should raise suspicion for Andersen–Tawil syndrome, prompting triad assessment and consideration of genetic testing.
- Clinical presentation of Andersen–Tawil syndrome shows great variability, and dysmorphic features may be overlooked due to subtle or indistinct changes.

## Introduction

Andersen–Tawil syndrome (ATS) is a rare genetic disorder with a reported prevalence of 1:1 000 000.^1^ It is caused by a loss-of-function mutation in the *KCNJ2* gene, which affects the function of the inward rectifying potassium channel *Kir2.1*.^2,3^

Patients with ATS typically present with a symptom triad of electrocardiographic abnormalities (nearly 100% penetrance), periodic muscular weakness/paralysis (35%), and distinctive dysmorphic manifestations (75%, most commonly small mandible).^4^ Strong genotype–phenotype correlation exists, but clinical presentation varies even within families.^5,6^ This makes diagnosis challenging. Yet, achieving diagnosis is important since patients with ATS face a high risk of ventricular arrhythmias.^4,7^ With appropriate management, such as beta-blockers, potassium-sparing diuretics, and placement of an implantable cardioverter-defibrillator (ICD), they can achieve a good prognosis.^7,8,9^

This case report aims to create awareness of ATS and demonstrate the importance of comprehensive diagnostic work-up.

## Summary figure

**Figure:**
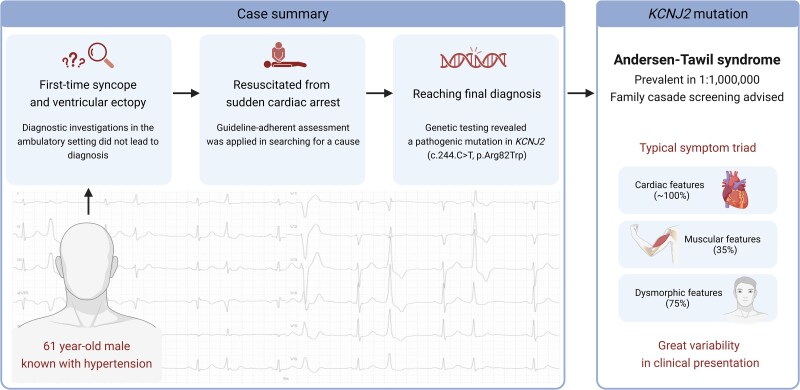


## Case presentation

**Figure 1:**
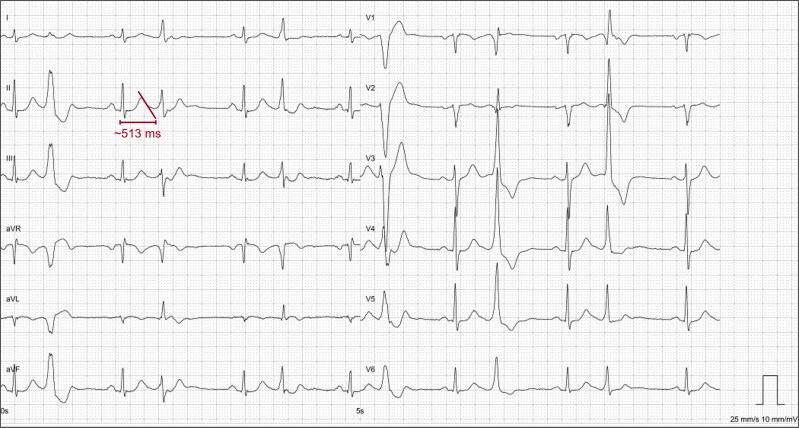
Baseline electrocardiogram from the first hospital admission showing multifocal PVCs in bigeminy partly arising from the basal left ventricular septum, possibly even from the his region, and the right ventricular outflow tract. The QTc interval was >500 ms. QTc was later normalized.

We report on a 61-year-old hypertensive male admitted to a local hospital following his first syncopal episode while walking. He experienced involuntary micturition during unconsciousness but otherwise denied prodromes or persisting symptoms. The clinical assessment was inconspicuous, aside from the electrocardiogram and telemetry revealing excessive, multifocal ventricular ectopy that intermittently presented in bigeminy (*Figure 1*). The QTc interval was prolonged >500 ms using Bazett's formula in one ECG shortly after admission, potentially confounded by frequent premature ventricular contractions (PVCs).

**Figure 2:**
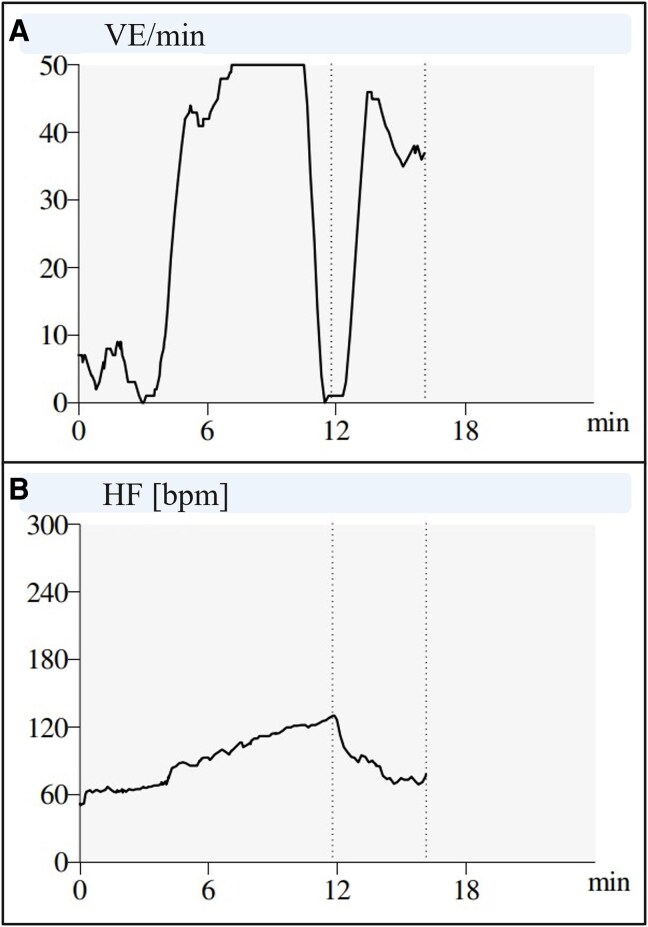
Exercise testing results: *(A)* shows the amount of the ventricular ectopy per minute (VE/min); *(B)* shows the patient’s heart rate by beats per minute [HR (b.p.m.)]. Note how the ectopy ceases at approximately 11 min, where the patient’s heart rate surpasses 120 b.p.m.

**Figure 3:**
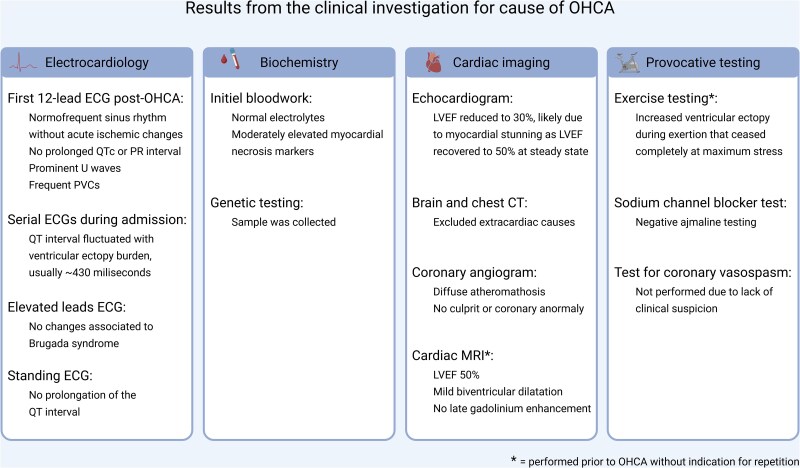
Test results following ESC guidelines on ventricular arrhythmias and the prevention of sudden cardiac death. Abbreviations: CT, computed tomography; ECG, electrocardiogram; LVEF, left ventricular ejection fraction; MRI, magnetic resonance imaging; OHCA, out-of-hospital cardiac arrest; PVCs, premature ventricular contractions. Created in BioRender. Stavnem, D. (2026) https://BioRender.com/novtgym

Before discharge the following day, the patient was prescribed beta-blocker therapy (metoprolol 50 mg/day) and referred for further cardiac evaluation: Holter monitoring confirmed polymorphic ventricular ectopy of 20% (12 274 PVCs/24 h). Ectopy, predominantly right ventricular outflow tract origin, increased during exercise testing yet ceased at maximum stress (*Figure 2*). Comprehensive cardiac imaging revealed mild abnormalities including biatrial and biventricular dilatation, borderline-reduced left ventricular ejection fraction (50%), no fibrosis on magnetic resonance imaging, and slightly inhomogeneous left ventricular perfusion on Rubidium-48 myocardial perfusion imaging (computed tomography was inconclusive due to frequent PVCs). With no definitive diagnosis established, the patient was referred for an electrophysiological study (EPS) with a potential PVC-ablation and implantable loop-recorder insertion. However, seven months after initial presentation and while awaiting these procedures, the patient collapsed with sudden cardiac arrest whilst bicycling. Bystanders immediately initiated cardiopulmonary resuscitation, and paramedics provided standardized advanced revival. The primary rhythm was ventricular fibrillation, and external defibrillation was delivered four times. Return of spontaneous circulation was achieved after 17 min. The patient regained consciousness and was transported to a tertiary cardiac arrest centre where investigations according to the European Society of Cardiology’s (ESC’s) ‘Algorithm for the evaluation of sudden cardiac arrest survivors’ was initiated^10^: The results from this diagnostic work-up is summarized in *Figure 3*. The tentative diagnosis was arrhythmogenic cardiomyopathy. To confirm, genetic testing was performed.

The patient did not experience adverse events during hospitalization. He received an ICD but was not prescribed additional medicine. He was discharged after 11 days. He recovered fully without any cognitive deficits and returned to his work fulltime only a few weeks later.

Upon discharge, he was referred to the outpatient unit for hereditary heart diseases for a follow-up consultation, and the planned EPS was conducted 2 months later. Despite PVCs being present during the EPS, activation mapping revealed a deep, septal focus not accessible for endocardial ablation.

During follow-up, the genetic testing revealed a pathogenic mutation in the *KCNJ2* gene (c.244.C>T, p.Arg82Trp), associated to ATS.^11^ However, the patient did not appear to have the characteristic muscular or dysmorphic features. Since *KCNJ2* mutations can be inherited in an autosomal dominant manner, genetic testing within the family was recommended.^12^ Numerous family members tested positive for the mutation and presented a cardiac phenotype (frequent PVCs and/or presence of U waves) but had no obvious dysmorphic manifestations. Eventually, one relative remarkably disclosed having webbed toes and reported that this trait was common among family members. This was regarded as a dysmorphic feature, which consequently supported the final diagnosis of ATS.

Upon achieving diagnosis, the case patient’s pharmacological treatment was changed in accordance with guidelines on managing ATS.^10^ He was advised to avoid QT-prolonging drugs and switched to a non-selective beta-blocker (propranolol 80 mg/day). This led to significant ectopy reduction from 20% to 6%, and after doubling the dosage, the PVCs fell to <1%. He tolerated his medicine well and has remained asymptomatic without adverse events or appropriate ICD therapy.

## Discussion

According to the ESC guidelines on ventricular arrhythmias and prevention of sudden cardiac death, ATS should be considered in patients without structural heart disease presenting with ≥2 of: prominent U waves ± QT prolongation, bidirectional/polymorphic PVCs/ventricular tachycardia, dysmorphic features, periodic paralysis, and/or *KCNJ2* mutation.^10^

Given the absence of dysmorphic features and paralysis, yet the presence of minor structural abnormalities and exertional syncope/cardiac arrest, the initial differential diagnoses reasonably included both cardiomyopathy and primary electrical diseases. Among the latter, catecholaminergic polymorphic ventricular tachycardia (CPVT) was considered. However, several features were not typical for CPVT: the onset in adulthood rather than childhood or adolescence, the high resting ectopy burden, and abnormal echocardiographic findings.^5,12^ The exercise testing results also revealed an important distinction, as cessation of ventricular ectopy at peak exercise is common in ATS, whereas it typically increases in CPVT, though neither finding is pathognomonic.^13^

Although ATS is classified as long QT syndrome type 7, research proposes that this may be misleading.^3,4^ A study in patients with *KCNJ2* mutations demonstrated only modest QT prolongations and suggested that U waves were mistakenly included in the QT measurements.^3^ In ATS, U waves are characteristically prominent with a prolonged terminal T wave downslope and broad T-U junction, which complicates accurate QT measurement.^3^ No consistent QT prolongation was observed in our patient. Regardless, pharmacological treatment of ATS still follows established principles for channelopathy management^1,4^: QT-prolonging drugs including amiodarone should be avoided, while guidelines recommend considering non-selective beta-blockers (preferably nadolol or propranolol) and/or flecainide.^10^ However, flecainide use remains debated, as studies suggest only partial efficacy and possibly proarrhythmic effect.^4,9,14^ A history of unexplained syncope has been associated with a more serious phenotype with an increased risk of life-threatening arrhythmic events,^4^ which this case demonstrates. Although beta-blockers reduced ectopy burden in our patient, research has questioned their protective effect against life-threatening arrhythmias in ATS.^4^

While this case presented significant diagnostic challenges, it reinforces the critical role of complete guideline-adherent investigation, particularly emphasizing the value of genetic testing. The subtle dysmorphic feature supporting the definitive diagnosis underscores the importance of family screening, which further enables early detection, risk stratification, and arrhythmia management in relatives, potentially preventing adverse events.^12,15^

In conclusion, comprehensive diagnostic work-up including genetic testing following out-of-hospital cardiac arrest is critical in reaching diagnosis in rare cases like ATS.

## Lead author biography


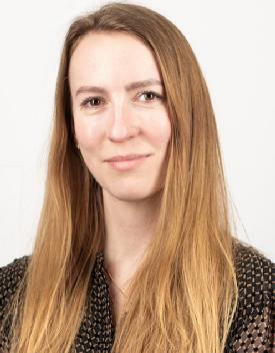


Dorte Stavnem is a physician and PhD candidate in the Department of Cardiology, Copenhagen University Hospital, Rigshospitalet, Denmark. Her research focuses on the clinical evaluation and management of sudden cardiac arrest survivors, including risk stratification, arrhythmia prevention, and genetic cardiovascular disorders.

## Data Availability

The data underlying this article will be shared on reasonable request to the corresponding author.
